# Pott’s Puffy Tumor, a Forgotten Complication of Sinusitis: Report of Two Cases

**DOI:** 10.7759/cureus.33452

**Published:** 2023-01-06

**Authors:** Elnaz Jalali, Anusha Vaddi, Kandasamy Rengasamy, Aditya Tadinada

**Affiliations:** 1 Oral and Maxillofacial Radiology, Oral and Maxillofacial Radiology Private Practice, Dallas, USA; 2 Oral and Maxillofacial Radiology, Virginia Commonwealth University School of Dentistry, Richmond, USA; 3 Oral and Maxillofacial Radiology, University of Connecticut Health Center, Farmington, USA

**Keywords:** hemi pansinusitis, osteomyelitis of frontal bone, frontal sinusitis, pott’s puffy tumor, frontal sinus

## Abstract

Pott’s puffy tumor (PPT) is a life-threatening complication of infectious sinusitis/osteomyelitis of the frontal bone. It occurs due to infection or trauma and is often seen in late childhood and adolescence. If left untreated for a protracted period, intracranial complications such as epidural abscess, subdural empyema, meningitis, and a cerebral abscess may occur. The diagnosis is often confirmed with CT. Prompt medical and surgical treatment is mandatory as there is the potential for significant morbidity if not quickly diagnosed and treated. This paper presents two cases of PPT manifested in patients with hemi pansinusitis.

## Introduction

Pott’s puffy tumor (PPT) was first described by Sir Percivall Pott in 1760 [1]. Although named as a tumor, it is not a true neoplasm, mainly infectious in origin. It occurs as a sequela of frontal sinusitis or trauma, progressing as osteomyelitis of the frontal bone with associated subperiosteal abscess. PPT is predominantly reported in the childhood and adolescent age group. The presenting clinical symptoms include headache, swelling of the forehead, orbital swelling, chills, fever, and rhinorrhea [2,3]. The diagnosis is often confirmed with CT. Prompt medical and surgical intervention is necessary as there is potential for the spread of infection into the cranium, causing intracranial complications such as epidural abscess, subdural empyema, meningitis, and cerebral abscess [3,4]. We report two cases of PPT presented with hemipansinusitis. This case report was previously presented as a meeting abstract at the 2014 American Academy of Oral and Maxillofacial Radiology meeting.

## Case presentation

Case report 1

**Figure 1:**
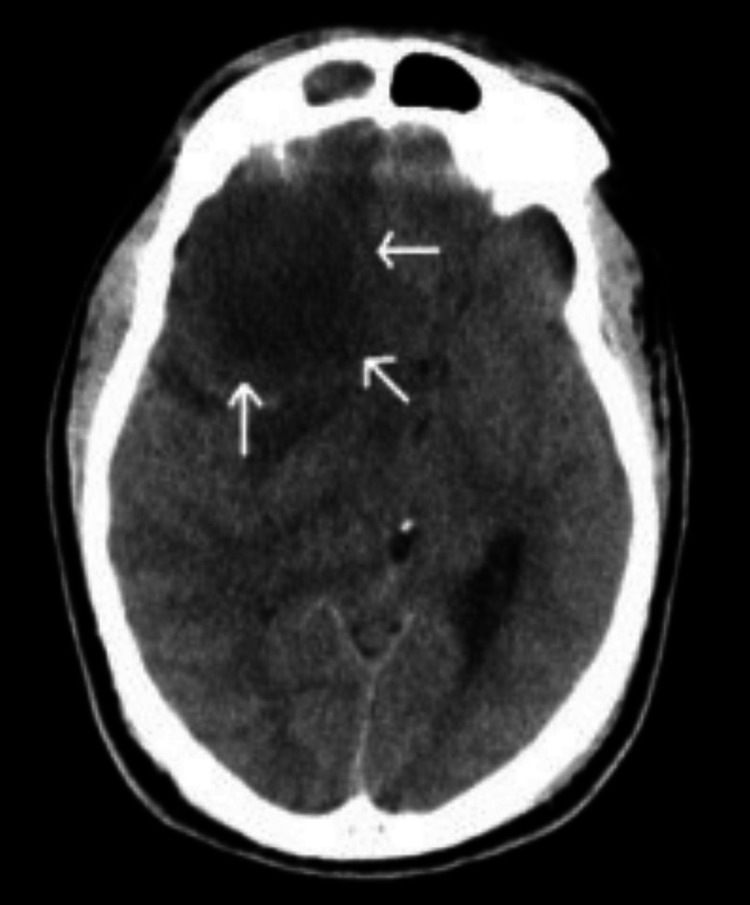
Axial CT (soft-tissue window) demonstrating hypodense right frontal lobe mass with hyperdense rim (arrows).

**Figure 2:**
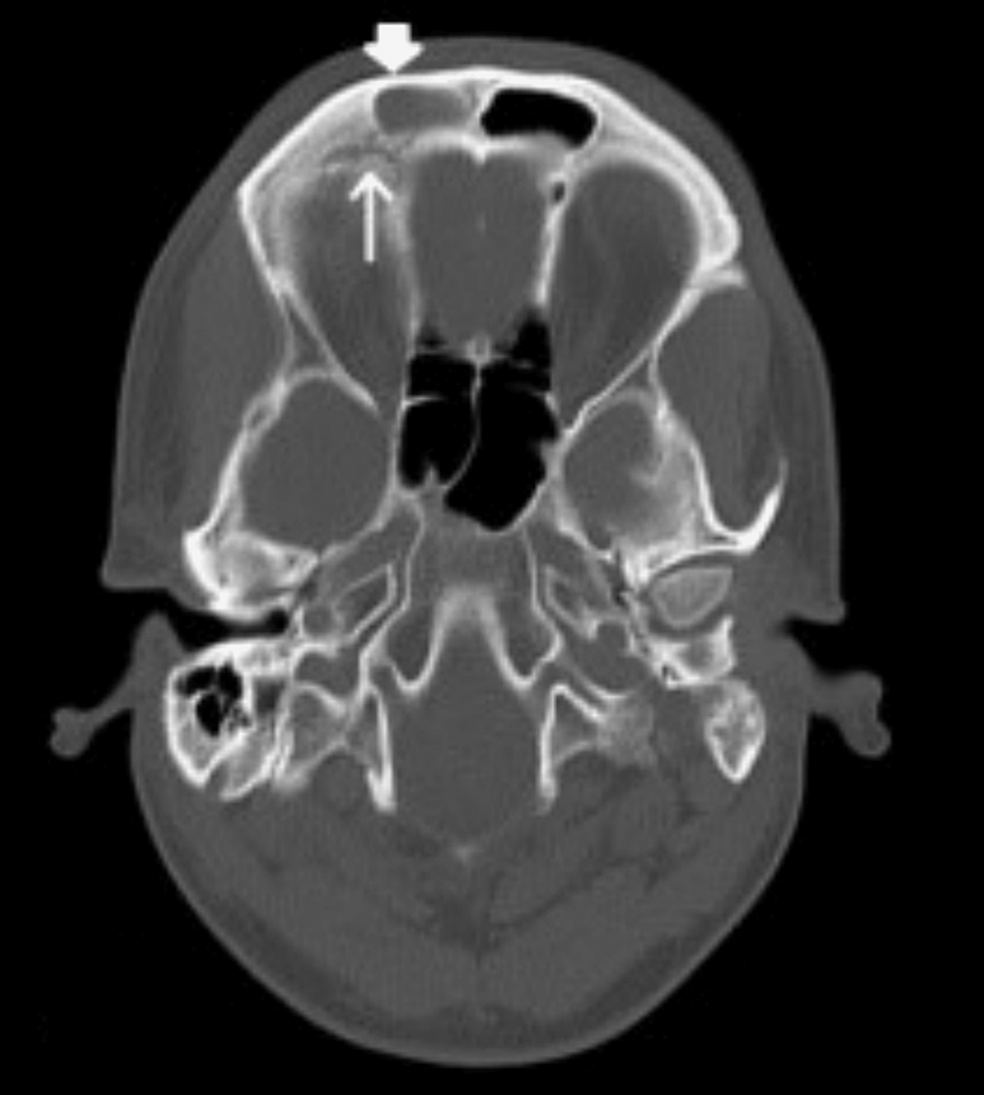
Axial CT without contrast demonstrating erosion of the posterior table of the right frontal sinus (arrow) and right frontal sinus opacification (arrowhead).

**Figure 3:**
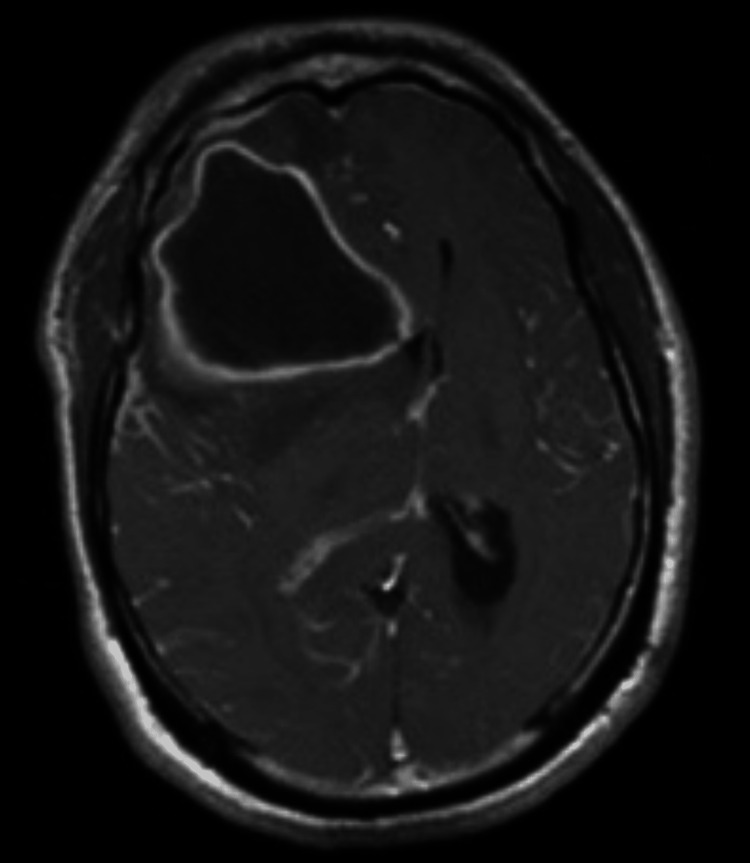
T1-weighted post-gadolinium demonstrating right frontal lobe mass, hypointense center with a thin enhancing rim.

**Figure 4:**
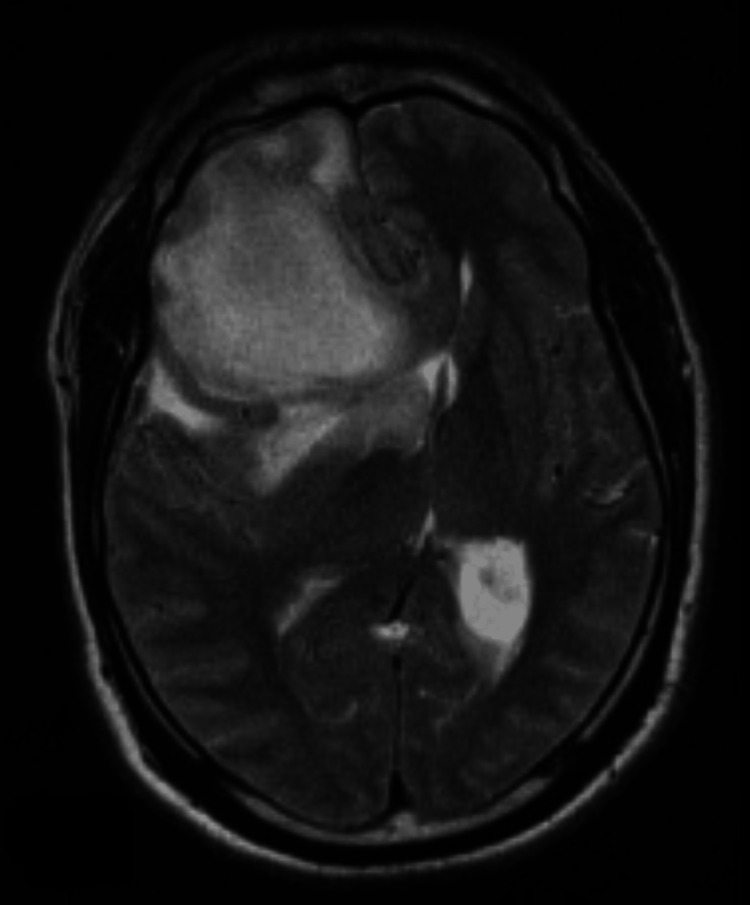
T2-weighted images demonstrating hyperintense mass. There is moderate vasogenic edema in the right frontal lobe and severe mass effect with right-to-left midline shift.

**Figure 5:**
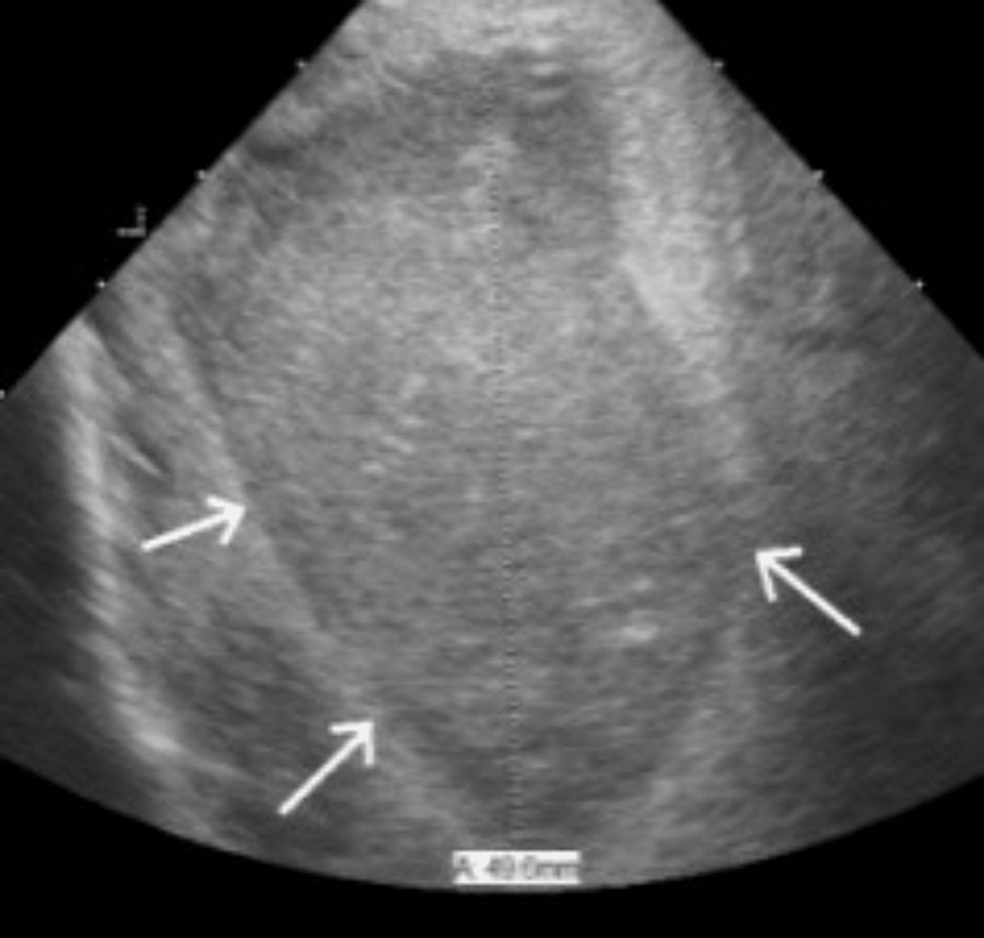
Intraoperative-focused ultrasound of right frontal lobe demonstrating frontal lobe abscess (arrows) from frontal sinus disease, measuring 5 cm in diameter.

**Figure 6:**
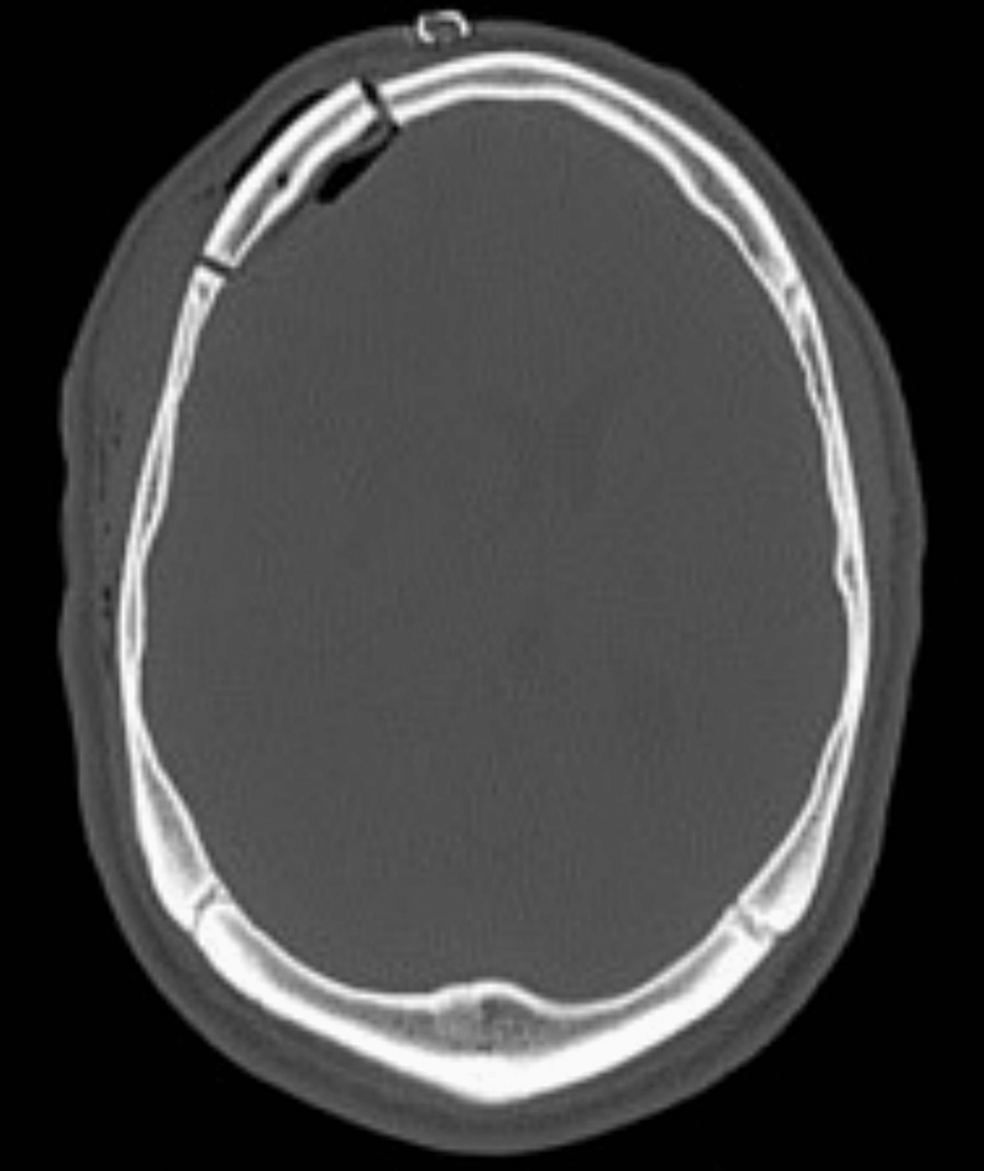
Axial CT demonstrating postsurgical changes after right frontal craniectomy.

A 28-year-old man presented to the Department of Neuroradiology, UConn Health, with complaints of left-sided extremity numbness, altered mental status, and swelling of the forehead on the right side (supraorbital region). CT demonstrated a large right frontal lobe mass with vasogenic edema consistent with a cerebral abscess. The mass and edema compressed the adjacent portion of the right lateral ventricle and caused an approximately two cm right-to-left midline shift. There was partial opacification of the right frontal sinus with erosion through the anterior wall of the sinus (Figures 1-2). Also, there were right hemipansinusitis involving ethmoidal air cells and maxillary sinus. MRI confirmed the findings of the CT scan (Figures 3-4). A right frontal craniectomy was performed. Intraoperative focused ultrasound of the right frontal lobe demonstrated a frontal lobe abscess of 5 cm in diameter (Figure 5). The right frontal lobe abscess was removed. Immediate postoperative CT demonstrated air within the frontal lobe consistent with the evacuation of prior abscess collection. The residual gas in the abscess cavity extended along the operative tract into subcutaneous soft tissues (Figure 6). The midline shift decreased from 1.5 to 1 cm. Periodic follow-up using CT and MRI scans demonstrated improvement in mass effect from the residual abscess and midline shift. In the region of vasogenic edema, there was mild parenchymal enhancement at the gray-white junction. In addition, interval improvement in aeration of the right ethmoidal and maxillary sinus with a small amount of residual mucosal thickening of the maxillary sinus was noted.

Case report 2

**Figure 7:**
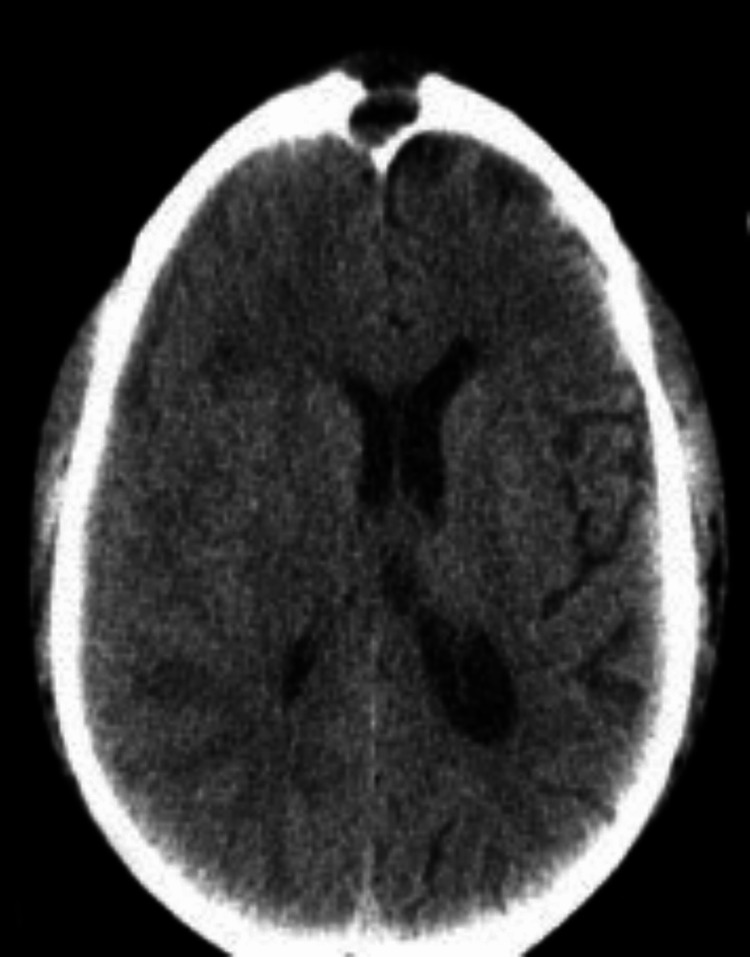
Axial CT without contrast, demonstrating erosion of walls of the frontal sinus.

**Figure 8:**
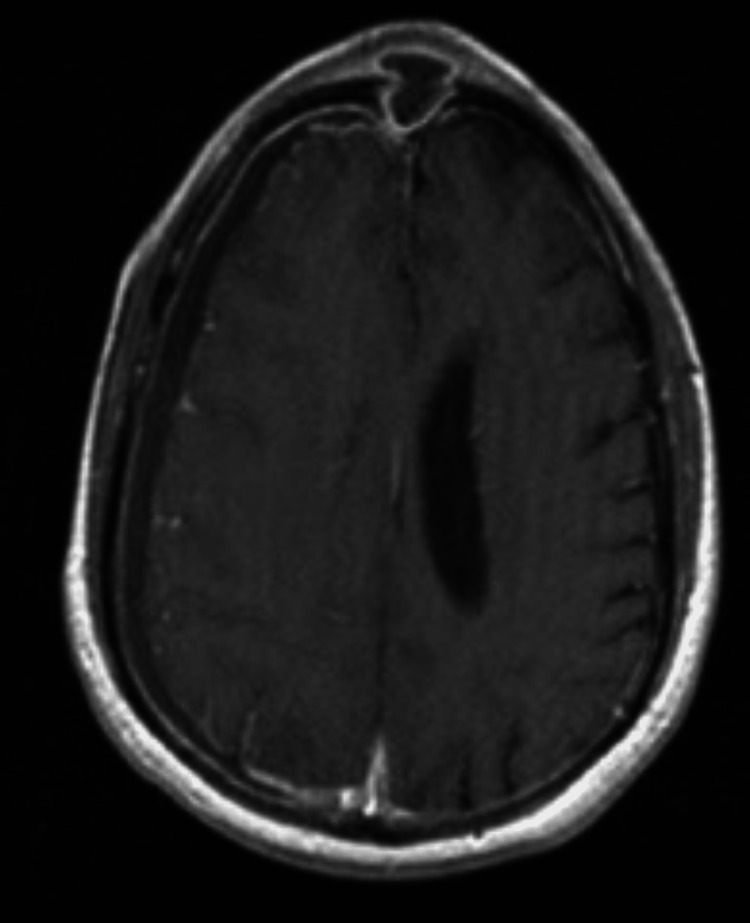
Axial T1-weighted post-gadolinium demonstrating hypointense fluid collection with an enhancement of walls centered at the frontal sinus and extending subcutaneously.

**Figure 9:**
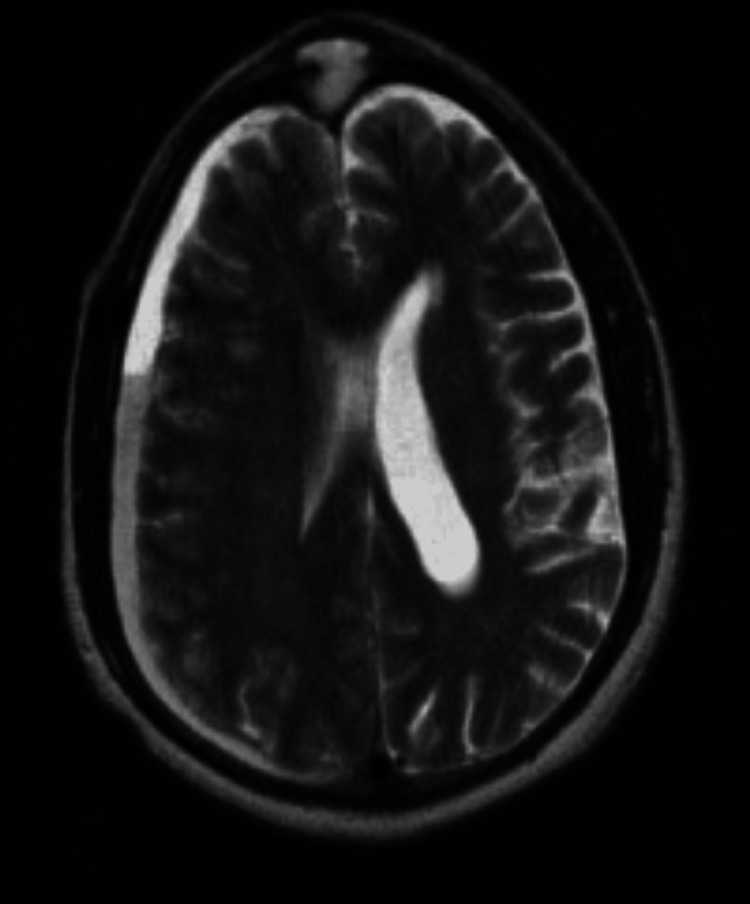
Axial T2-weighted image demonstrating subdural collection with layering effect likely due to settled inflammatory debris or hemorrhage.

**Figure 10:**
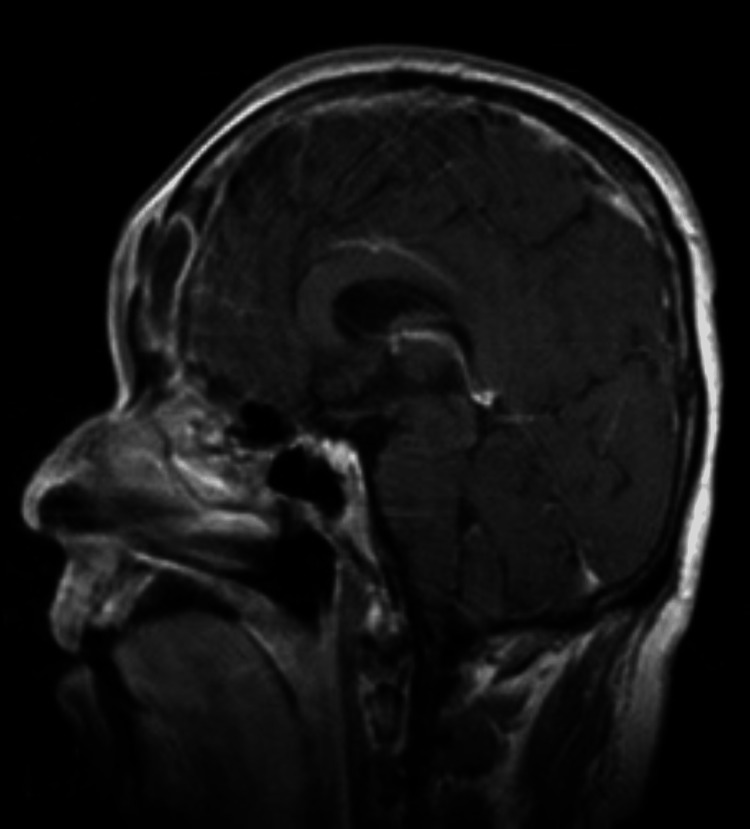
Sagittal T1-weighted post-gadolinium images demonstrating destruction in the interim anterior and posterior walls with intracranial extension as well as extension into the extracranial soft tissues of the frontal region. Fluid collection within the subdural space is also noted (subdural empyema).

**Figure 11:**
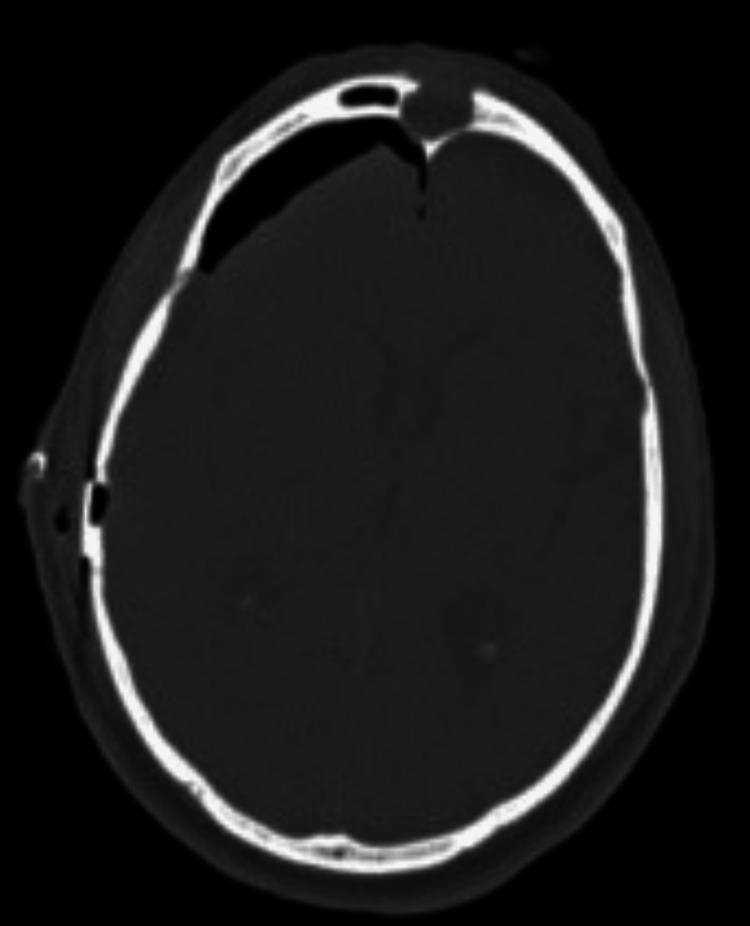
Postoperative (Day 1) image demonstrates the gas within the abscess cavity extending along the operative tract into the subcutaneous soft tissues.

**Figure 12:**
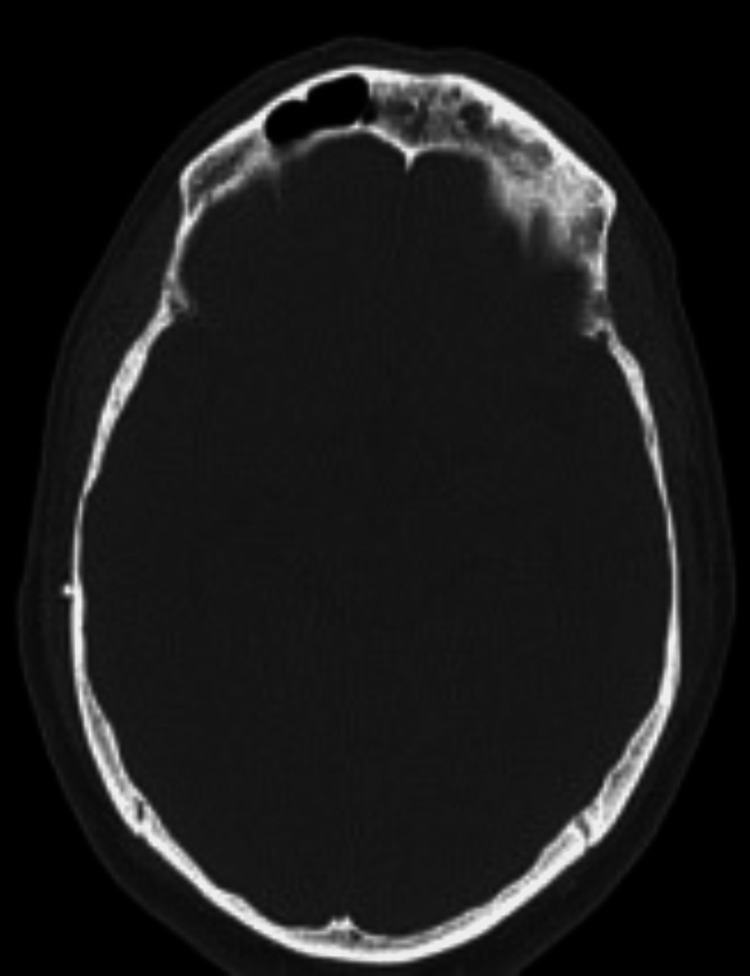
A 16-month postsurgical axial CT demonstrating progressive ossification of the left frontal sinus.

A 54-year-old man presented to the Department of Neuroradiology, UConn Health, with a history of sinusitis and a chief complaint of severe frontal headache with progressive swelling in the center of the forehead. CT scan revealed bony erosion of the anterior and posterior walls of the right frontal sinus (Figure 7). Hemipansinusitis involves the left maxillary sinus, frontal sinuses, right maxillary sinus, and a few ethmoidal air cells. MRI showed a subdural empyema measuring approximately 9 mm in diameter and a 6-7 mm midline shift to the left and right lateral ventricle compression due to mass effect (Figures 8-10). Right parietal craniotomy was performed to drain subdural empyema. One-day postoperative CT demonstrated pneumocephalus (Figure 11). A small amount of fluid is collected laterally to the right cerebral hemisphere. An approximate 3 mm shift of midline structures to the left was done. Periodic follow-up scans revealed a gradual improvement in pneumocephalus and midline shift. A six-month post-op scan demonstrated bone growth along the margins of the left frontal sinus, decreasing the volume of the sinus. There was no direct communication from the frontal sinus to the cranial compartment. A 16-month postoperative CT demonstrated progressive ossification of the left frontal sinus. Also, there was no focal mass, midline shift, abnormal enhancement, or acute intracranial hemorrhage (Figure 12). The findings of CT provided the key to the diagnosis in both cases. The combination of the sinusitis and cerebral abscess or subdural empyema suggested that abscesses are secondary to frontal sinusitis.

## Discussion

PPT is a classical sign of frontal sinus osteomyelitis. Although considered rare in the post-antibiotic era, PPT is still a life-threatening complication due to frontal sinus infection. The pathogenesis is due to the spread of infection/sinusitis to the frontal bone. Percival Pott postulated that a puffy, circumscribed, indolent scalp tumor due to dural inflammation and spontaneous separation of pericranium from the skull occurs due to head injury [1]. Later in 1879, Lannelongue recognized the infectious origin. Tilley and Luc in 1899 found cranial osteomyelitis might occur as a complication of PNS infection [3]. Raja V et al. reported a case of preorbital cellulitis following an insect bite that progressed to PPT [5]. Although the majority of cases were reported in childhood and adolescence, PPT can occur in any age group [2,4,6,7]. In the present study, the patients were middle-aged adults (28 years, 57 years). The frontal sinus begins forming around two years of life, radiographically visible around six years, and growth of the sinus continues through puberty [8]. During early teens, the frontal sinus reaches adult size, and there is increased vascularity of diploic veins. The communication between trabecular bone and sinus mucosa during this age favors the development of osteomyelitis [7]. In middle-aged adults or the elderly group, potential predisposing factors such as trauma and frontal sinus infection lead to the establishment of aforementioned communication and the development of PPT.

In both cases, untreated frontal sinusitis for a prolonged period led to intracranial complications. A radiographic examination of both patients revealed hemipansinusitis. Mittal MK et al. reported a case of pansinusitis progressing as meningitis with intracranial complications [9]. This emphasizes the importance of prompt diagnosis and intervention. If the pansinusitis and associated swelling of the forehead occurred due to trauma or infection, irrespective of age group, PPT must be considered as a possible differential diagnosis. On the other hand, if pansinusitis was noted in children without any pre-existing cause, cystic fibrosis (CF) should be considered as one of the differential diagnoses. However, in most cases, CF can be diagnosed clinically based on symptoms related to impaired nasal secretions and exocrine gland insufficiency. Imaging aids in determining the extent of the sinonasal disease [10].

If a patient presents with swelling of the forehead, osteomyelitis of the frontal bone should be suspected, and CT and MRI imaging should be performed. Radiographic differential diagnosis of Potts puffy tumor is destructive metastasis to the frontal sinus and unilateral non-Hodgkin’s lymphoma. The presence of underlying frontal sinusitis and osteitis of sinus walls in scans helps differentiate PPT from the aforementioned conditions. In challenging cases, bone scintigraphy can be performed [11].

In recent times, Cone-beam computed tomography (CBCT) has been widely employed for diagnosis and treatment planning in the oral and maxillofacial region. Some of the large field-of-view CBCT scans can capture all the paranasal sinuses, including the frontal sinus. Therefore, if signs of chronic pansinusitis or hemipansinusitis are evident, along with other bony changes, it warrants further investigation and referral to the concerned medical specialist.

Once the diagnosis is established, depending on the severity of complications, treatment of PPT ranges from conservative broad-spectrum antibiotics to surgery. Antibiotics are prescribed to patients as soon as the diagnosis is made to prevent neurologic sequala. In some cases, culture is performed to determine the source of infection, and antibiotics are prescribed based on the micro-organism. Surgical intervention depends on the disease course progression. In some cases, frontal craniotomy is performed, followed by subdural empyema and subperiosteal abscess drainage. Functional endoscopic sinus surgery is performed in a few cases [12]. Since both patients had intracranial complications, surgery was chosen as the treatment of choice. Periodic follow-up with imaging examination was performed in both cases till the pathology was resolved.

## Conclusions

Pott’s puffy tumor is a potentially life-threatening complication of chronic frontal sinusitis. This paper presents two cases of chronic frontal sinusitis with intracranial complications. Both cases were managed by surgical intervention. Periodic follow-up scans showed resolution in both patients. These reports highlight the utmost importance of early diagnosis and intervention for a seemingly benign infection like bacterial sinusitis. Oral and maxillofacial radiologists should be aware of this condition since the frontal sinus and bones are imaged in most of the large-volume CBCT scans. Early diagnosis and intervention of this condition help in avoiding fatal neurological complications.
